# Parental Influences on Pathogen Resistance in Brown Trout Embryos and Effects of Outcrossing within a River Network

**DOI:** 10.1371/journal.pone.0057832

**Published:** 2013-02-22

**Authors:** Emily S. Clark, Rike B. Stelkens, Claus Wedekind

**Affiliations:** 1 Department of Ecology and Evolution, University of Lausanne, Lausanne, Switzerland; 2 Institute of Integrative Biology, University of Liverpool, Liverpool, United Kingdom; Biodiversity Insitute of Ontario-University of Guelph, Canada

## Abstract

Phenotypic plasticity can increase tolerance to heterogeneous environments but the elevations and slopes of reaction norms are often population specific. Disruption of locally adapted reaction norms through outcrossing can lower individual viability. Here, we sampled five genetically distinct populations of brown trout (*Salmo trutta*) from within a river network, crossed them in a full-factorial design, and challenged the embryos with the opportunistic pathogen *Pseudomonas fluorescens*. By virtue of our design, we were able to disentangle effects of genetic crossing distance from sire and dam effects on early life-history traits. While pathogen infection did not increase mortality, it was associated with delayed hatching of smaller larvae with reduced yolk sac reserves. We found no evidence of a relationship between genetic distance (*W*, F_ST_) and the expression of early-life history traits. Moreover, hybrids did not differ in phenotypic means or reaction norms in comparison to offspring from within-population crosses. Heritable variation in early life-history traits was found to remain stable across the control and pathogen environments. Our findings show that outcrossing within a rather narrow geographical scale can have neutral effects on F_1_ hybrid viability at the embryonic stage, i.e. at a stage when environmental and genetic effects on phenotypes are usually large.

## Introduction

The ability for a genotype to adopt different phenotypes according to environmental conditions permits organisms to maximize fitness in the face of heterogeneous biotic and abiotic risks [1]. As natural populations are increasingly exposed to habitat-altering anthropogenic activities [2], [3], this plasticity can be a key factor in increasing tolerance to environmental change [4]. The capacity of such traits to evolve in a population relies on the persistence of heritable variation therein [5]. While studies in the laboratory and the wild have demonstrated significant genetic variation in both trait means and reaction norms in plants [6], birds, [7], fish [8], [9], and amphibians alike [10], [11], environmental stress can reduce this heritable variation [12], [13]. Notably, the impact of stress on trait heritability is not always negative, with studies also showing that genetic variance can increase [11], [14], [15], or remain stable [16], [17], [18] across environments.

Taken together, there appears to be no clear consensus on how environmental change affects genetic variability in quantitative traits, with indications that it is not only trait-dependent, but also reliant on the stressor in question. To add an additional level of complexity, the effect of ecological stressors on the genetic variability of plastic traits cannot be generalized for a species. Rather, populations often diverge in the genetic architecture of traits and trait plasticities, as a result of selective pressures varying in type and intensity over space and time [for examples see 19], [20]. This divergence can indicate local adaptation [21], and as such, changes to the elevation and/or slopes of the reaction norms through outbreeding can have negative effects on fitness. Events including interpopulation hybridization have been shown to bring about such alterations [22], [23], [24].

We here investigate how outcrossing in a salmonid population network affects the expression and plasticity of early life-history traits. Outcrossing occurs more commonly in fish than any other vertebrate taxa [25]. It frequently arises in salmonids because of introgression from farmed stock into wild populations [23], [26]. Introgression from domestic stock can cause management complications as populations may suffer from lower fitness due to outbreeding depression, i.e. the breakdown of local adaptation and genetic incompatibilities [27], [28]. Although fitness declines are particularly evident in F_2_ and later generations, as co-adapted parental gene combinations are disrupted by recombination, they can also manifest in F_1_ hybrids [e.g. 23], [29], [30], [31], [32]. Hybrid breakdown in the first generation may be attributable to both extrinsic and intrinsic processes, including modifications to gene-by-environment interactions (local adaptation), underdominance, and/or epistasis [33]. At the same time, increased gene flow can have heterotic effects, especially between inbred populations, and the increased genetic variation generated by hybridization may indeed be instrumental in saving threatened populations from inbreeding depression [34]. Thus, whether populations benefit or suffer from outcrossing should generally depend on the genetic crossing distance, as fitness is expected to peak at intermediate genetic distance under the opposing effects of inbreeding and outbreeding depression [35], [36].

Populations of brown trout (*Salmo trutta*) are characterized by vast diversity in terms of both phenotype [37] and life history strategies [38]. They usually have complex structures, i.e. population differentiation within a confined area (e.g. a river catchment), and can show significant genetic and phenotypic divergence on microgeographic scales [39]. For our study, we conducted full-factorial *in vitro* fertilizations between five of the populations described in Stelkens et al. [39] yielding offspring from parents with varying genetic distances. We chose these populations because they are genetically significantly different using 11 microsatellite loci, (pairwise F_ST_ estimates between populations ranged from 0.005 to 0.035). Although global population differentiation in this system is moderate (global *F_ST_* = 0.022), significance of pairwise *F_ST_* comparisons is typical for salmonid populations within the same catchment [40], [41], [42], [43], [44]. In addition, the same five populations differ substantially in functional morphological traits (body and head shape), and average population phenotypes were found to match the flow regimes of their respective habitats [39]. This argues for the presence of local adaptation and/or phenotypically plastic responses to environmental heterogeneity. We used the offspring of these five populations to examine the relationship between genetic distance and phenotype and to assess whether between- and within-population crosses differ with respect to phenotypic means and norms of reaction.

Using pathogen challenges to investigate the fitness consequences of outcrossing has been suggested to be a highly effective method, as the intricate associations of genes in the immune system should be particularly susceptible to disruption [for discussion see 45]. Natural mortality at the embryonic stage can be significant and often seems pathogen-related [e.g. ref 46]. Moreover, environmental changes arising from human activities are predicted to result in increased occurrences of infectious disease [47], [48], [49], and have been linked to declines in salmonids [50]. We thus chose to simulate an ecologically relevant situation by inoculating a subset of embryos with the opportunistic fish pathogen, *P. fluorescens*, to measure the effect of infection on a suite of fitness-related traits. This bacterium is abundant in the aquatic environment [51], which is considered the principle reservoir of infection [52], and has been implicated in disease pathologies not only in adult fish [53], but also in whitefish [54], [55] and more recently in brown trout embryos [56]. Our design allowed us to disentangle variance components and compare the effects of outcrossing with sire and dam effects on offspring phenotype.

## Methods and Materials

### Ethics statement

Permissions for handling adults and embryos were granted by the Fishery Inspectorate of the Bern Canton. Additional authorizations from the cantonal veterinary office were not required as manipulations of the adults were part of the yearly hatchery program of the Bern Canton, and all experimental manipulations on embryos were performed prior to yolk sac absorption.

### Artificial fertilizations and rearing of brown trout embryos

**Figure 1:**
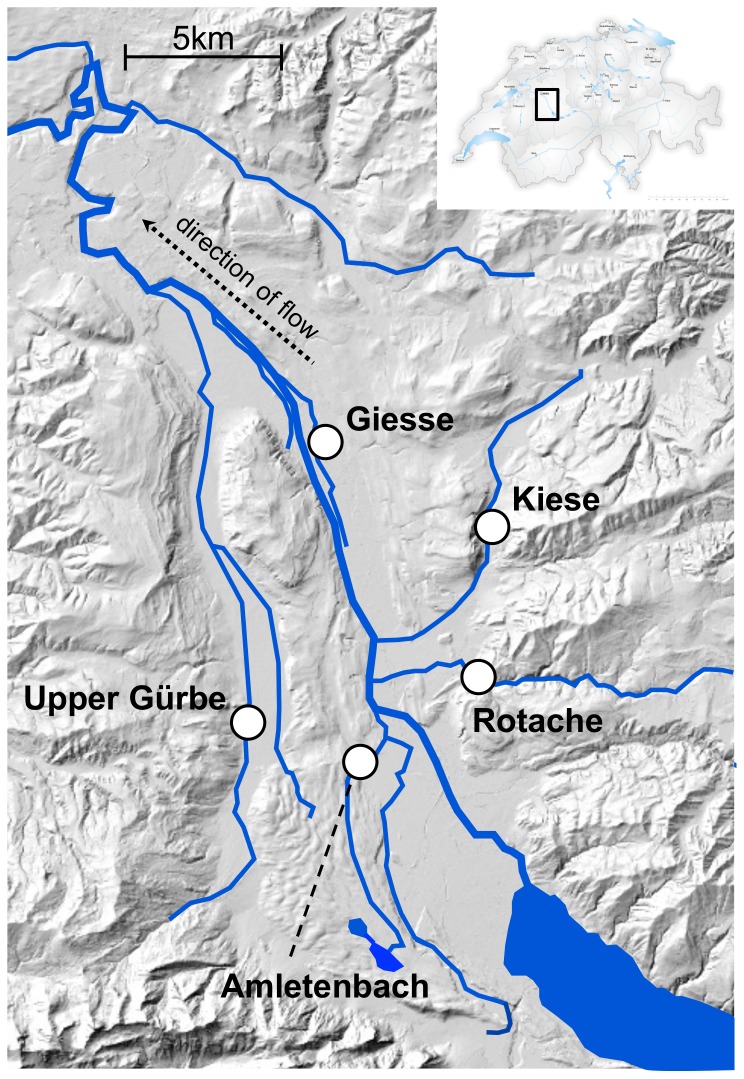
Locations of the five study populations in the Aare River catchment. Map adapted from Stelkens et al. 2012.

**Figure 2:**
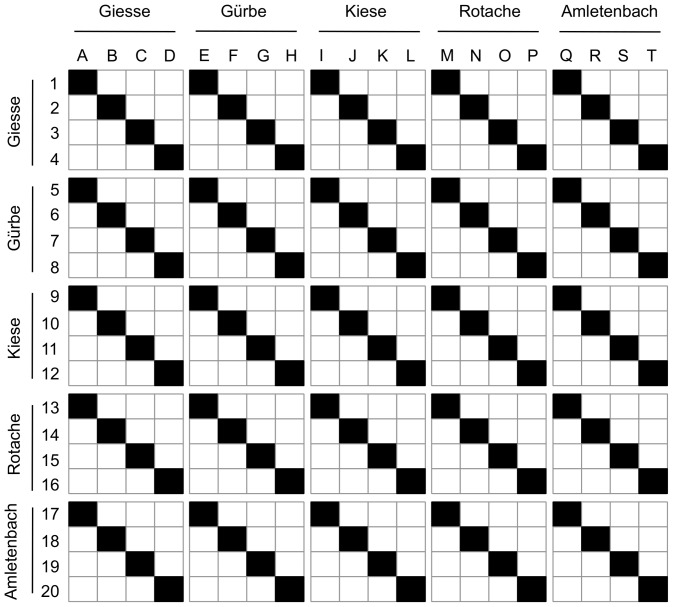
Breeding design. Females are indicated by letters, males by numbers, and crosses by black squares.

Adult brown trout were caught by electro-fishing from five tributaries of the river Aare (Figure 1) in the Canton of Bern, Switzerland shortly before spawning season in November 2009, and were kept in a hatchery near Reutigen until maturation. In previous years, brown trout from these five locations have been observed to mature around the same time under these hatchery conditions (U. Gutmann, unpublished observations). Therefore, all crossings could easily be performed on the same day. Four gravid females and four mature males from each of the five populations were randomly selected, weighed, measured (total length), photographed, and stripped of their gametes [following methods described in ref 57]. Gametes from the different populations were crossed full-factorially (with respect to population) in a block design (Figure 2). Specifically, five females (one from each population) were each crossed with five males from each tributary, and this was replicated four times (i.e. four blocks), yielding 100 full sibships. Resulting embryos were transported back to the laboratory, washed as described in von Siebenthal et al. [55], and distributed singly to 24-well plates (Falcon, Becton-Dickonson), filled with 2 ml of sterile standardized water per well [see guidelines in: 58]. Distributions were performed such that one plate contained eggs from all four females in one population, each crossed with males of the five populations. A set of five plates, therefore, contained all sibling combinations. This was replicated 21 times, resulting in 105 plates with 2100 embryos. Embryos were stored in a 6.5 °C climate chamber and were examined weekly for survival with a light table (Hama professional, LP 555) and a stereo zoom microscrope (Olympus SZX9) until the beginning of hatching, at which point they were monitored daily.

### Preparation of bacterial inoculum and infections

At 177 degree days (DD) post-fertilization, a strain of *P. fluorescens*, which was isolated from whitefish gills and had proven deleterious to their embryos (Clark et al. submitted), was inoculated into two flasks containing 100 ml of nutrient broth (5 g bacto-peptone and 3 g meat extract per liter of distilled water; Fluka Chemie). Cultures were incubated for 36 hours at 22 °C until reaching the exponential growth phase. Cultures were then pelleted at 4000 rpm and washed three times with sterile standardized water (ssH_2_O). Pellets were pooled and resuspended in 50 ml of ssH_2_O. Bacterial densities were calculated using a Helber counting chamber and examined at 400 X on a phase-contract microscope (Olympus CX41). The *P. fluorescens* culture was then diluted, so that an inoculation of 100μl would result in a concentration of 10^8^ bacterial cells per ml in each well. Nine embryos per sibship were treated with the bacterial solution, while another 12 embryos served as controls, and accordingly received 100 µl of sterile standardized water.

### Measurements of larval lengths and yolk sac volumes

Each larva was photographed on the day of hatching with an Olympus C-5060 and was subsequently measured in ImageJ (http://rsb.info.nih.gov/ij/). Standard length was measured, as well as the length and width of the yolk sac. The volume of the latter was calculated as in Jensen et al. [20]. Since not all pictures were of high quality and varied in resolution, the relative quality of the image (i.e. clarity as perceived by the measurer) was also scored as “high”, “medium”, or “low”. Image quality was then added as a random effect (factor with three levels) into statistical models examining variation in hatchling size or yolk sac volume (see below).

### Genetic differentiation between populations and individual breeders

In October 2009, Stelkens et al. collected tissue samples from a total of 603 brown trout from 21 locations in the Aare river system, including 226 individuals (ranging between 35–63 per population) from the five tributaries studied here (Figure 1; sampling methods are described in [39]). They estimated population-level genetic distances using FSTAT 2.9.4 [59]. We extracted the pairwise F_ST_ values of the five populations used here. We also calculated a relatedness coefficient *W*, using the software MER3.0 [60], that describes the relatedness of two individual breeders based on their allelic similarity. F_ST_ and *W* were both tested as predictors of phenotype.

### Statistical analysis

Survival was analyzed as a binomial response variable in general linear mixed effect models (GLMM), and hatching age, hatchling size, and yolk sac volumes as continuous response variables in linear mixed effect models (LMM). All analyses were conducted in R [61] using the lme4 package [62]. Treatment, population cross type (factor with two levels, i.e. between or within-population), pairwise F_ST_ estimates (a total of 15, including 5 within- and 10 between-population crossing) [obtained from 39], and *W* were entered as a fixed effects, while sire, dam, sire x dam interactions, and crossing block were entered as random effects. For any model pertaining to hatchling size or yolk sac volume, image quality was also added as a random effect (i.e. factor with three levels), as it appeared to account for a significant part of variation in these measurements. To assess the importance of each effect, a reference model including all relevant terms was compared to a model lacking the term of interest. To investigate the importance of interactions, a model incorporating the interaction term was compared to the reference model. Akaike information criteria (AIC), which provides a measure of model fit and model complexity (lower values indicate a better fit to the data) and likelihood ratio tests (LRT) were used to compare model fits.

Variance components were extracted from the mixed effect models and used to calculate the components of phenotypic variation [63]. Assuming that epistatic effects are negligible, additive genetic variance (V_A_) can be calculated as 4 x the sire component of variation (V_S_). Dominance genetic variance (V_D_) was calculated as 4 x the sire x dam component. Maternal environmental variance was estimated as V_M_ = V_Dam_−V_S_. Residual variance included environmental variance as well as ½ V_A_ and ¾ V_D_ [64]. Narrow-sense heritability estimates were calculated as in Lynch and Walsh [63] and coefficients of additive genetic variation (CV_A_) as in Houle [65]. In the case of yolk sac measurements, the CV_A_ was divided by three to account for the dimensionality of the measurement [65]. To assess whether traits have the potential to evolve independently in two different environments, we tested for cross-environmental trait correlations (Pearson’s product moment correlation r) using paternal sibgroup means of time to hatching and larval length. Using the family-mean approach to estimate genetic correlations provides a conservative test of whether the correlation coefficient is significantly different from zero, with the added benefit that the correlation cannot exceed ±1 [for discussion see: 63]. Pearson’s product moment correlations were also used to examine the relationship between average egg size per female and mean yolk sac volume, larval length, and time to hatching per female sibgroup in each treatment. As six tests were performed to analyze the relationship between egg size and larval traits, a Bonferroni correction was subsequently performed, resulting in an alpha value of 0.008.

To examine the power that we had to detect differences between population cross types in the expression of larval traits in each treatment, and also to detect population cross type x treatment interaction effects, we performed multi-level power analysis using a simulation-based approach. Specifically, data were repeatedly simulated (1000 iterations) from the hypothetical distribution of our data. That is to say that for each iteration, random effect estimates were drawn from a normal distribution and response variables were recalculated. Simulations were modified to match the distributions of the actual data by incorporating intercepts, fixed effect coefficients, and random effect variance estimates from our data. Models either including or excluding population cross type as a fixed effect were then fit to each generated data set, and compared with LRT, as above. Likewise, models incorporating the interaction term were compared to those without it to test for the interaction effect. The proportion of times the LRT yielded a significant p-value was used as the estimate of power. This was performed separately for each trait.

## Results

**Figure 3:**
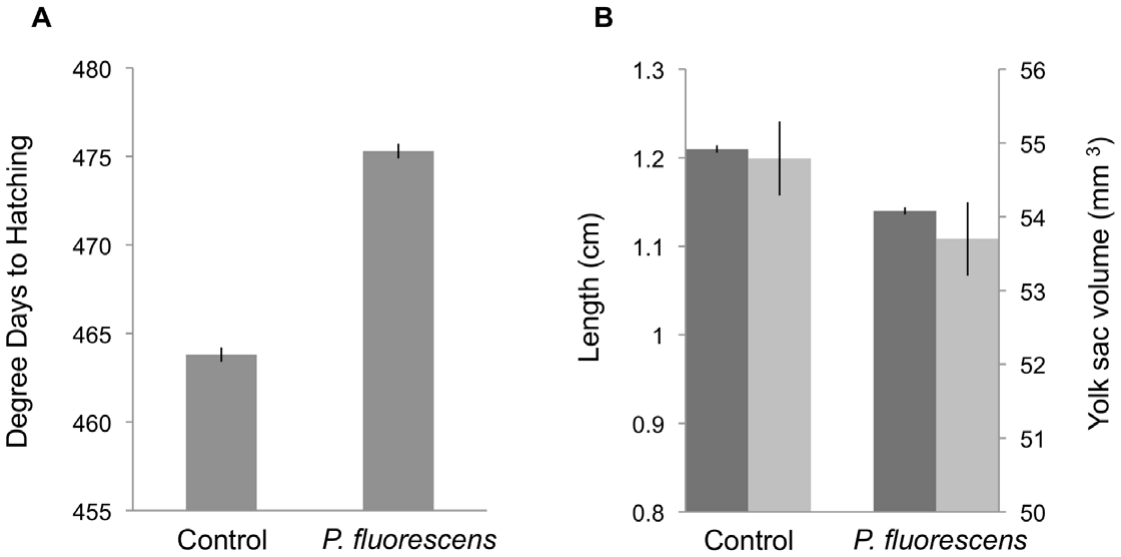
Mean time to hatching (A), larvae length, and yolk sac volume (B) (±SE) by treatment. In panel B, dark grey corresponds to larval length, and light grey to yolk sac volume.

Mean hatching success (±SE) in *P. fluorescens*-treated embryos (97.7±0.5%) was not significantly lower than the control (98.2±0.4%) (GLMM: Z = −0.5, p = 0.59). However, treatment did significantly delay hatching time and was associated with smaller larvae with reduced yolk sac volumes (Figure 3; Table 1).

**Table 1:** Likelihood ratio tests on mixed model logistic regression on plastic traits.

Model	Effect tested	AIC	X^2^	p
a) Hatching time				
**t+s+d+sxd**		15213		
s+d+sxd	t	15750	539.2	<0.001
t*p+s+d+sxd	t x p	15214	2.8	0.24
t+t|s+d+sxd	t x s	15203	14.1	<0.001
t+s+t|d+sxd	t x d	15182	35.3	<0.001
t+s+d+t|sxd	t x s x d	15188	29.0	<0.001
b) Hatchling length				
**t+s+d+sxd**		−3154.1		
s+d+sxd	t	−2962.8	193.3	<0.001
t*p+s+d+sxd	t x p	−3150.2	0.2	0.90
t+t|s+d+sxd	t x s	−3150.1	0.1	0.96
t+s+t|d +sxd	t x d	−3168.5	18.4	<0.001
t+s+d+t|sxd	t x s x d	−3150.1	0	1
c) Yolk sac volume				
**t+s+d+sxd**		9669.2		
s+d+sxd	t	9677.7	10.5	0.001
t*p+s+d+sxd	t x p	9669.3	3.9	0.14
t+t|s+d+sxd	t x s	9672.2	1.0	0.62
t+s+t|d+sxd	t x d	9672.7	0.5	0.78
t+s+d+t|sxd	t x s x d	9673.2	0	1

Reference models are indicated in bold. To test the effect of treatment, the reference model was compared to a model lacking treatment. For the other effects, the reference model was compared to a model incorporating the effect of interest. t: treatment; p: population cross type (within vs. between); s: sire; d: dam

**Figure 4:**
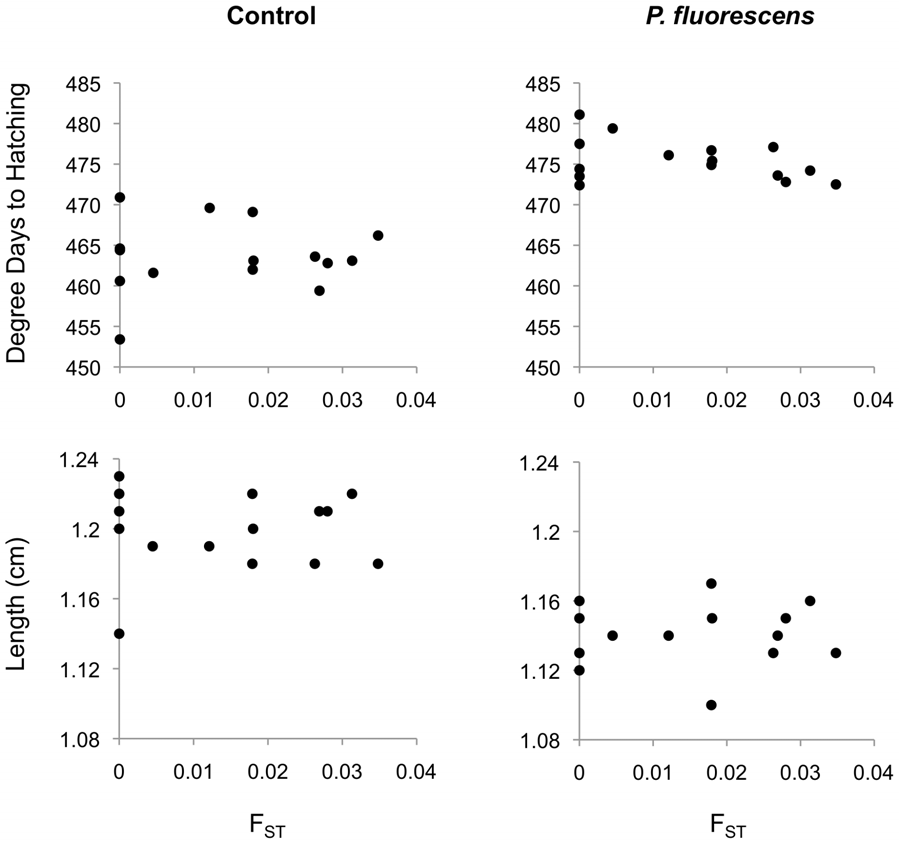
Genetic distance between population crosses (F_ST_) vs. hatching time and larval length in each treatment. Each point corresponds to least-square means for each population crossing distance (N = 15).

Incorporating F_ST_ as a linear predictor did not significantly improve the model fit in our analysis of hatching time or hatchling length in either the control or pathogen environments (Figure 4; Table 2). Adding either *W* as a linear predictor or F_ST_ as a quadratic predictor resulted in slight improvements of model fit only with respect to hatching time; however, when performing hypothesis testing, p-values of 0.05 and 0.04 (Table 2a) are considered weak indications of improved model fit [66, p. 96]. We therefore deferred to the more parsimonious model (i.e. to the reference model).

**Table 2:** Likelihood ratio tests on mixed model logistic regressions assessing the effects of genetic distance (F_ST_) and breeder relatedness (*W*) on hatching time and larval length in the two treatments.

Model	Effect tested	AIC	X^2^	p	AIC	X^2^	p
		Control			*P. fluorescens*		
a) Hatching time							
**s+d+s x d**		8769.7			6430.1		
F_ST_+s+d+s x d	F_ST_	8770.3	1.4	0.23	6430.2	1.9	0.17
F_ST_+F_ST_^2^+s+d+sxd	F_ST_^2^	8767.1	6.7	0.04	6432.2	2.1	0.35
*W*+s+d+sxd	*W*	8767.9	3.9	0.05	6431.8	0.2	0.63
b) Length							
**s+d+s x d**		−1716.3			−1421.4		
F_ST_+s+d+s x d	F_ST_	−1714.4	0.1	0.79	−1419.4	0	1
F_ST_+F_ST_^2^+s+d+sxd	F_ST_^2^	−1713.0	0.7	0.72	−1417.6	0.3	0.88
*W*+s+d+sxd	*W*	−1715.2	0.9	0.33	−1419.4	0	1

F_ST_ (as a linear or quadratic predictor) and *W* (linear predictor) were entered as fixed effects, while sire, dam, and sire x dam interaction effects were entered as random effects. Models incorporating F_ST_ or *W* were then compared to the reference models (indicated in bold). s: sire; d: dam.

Incorporating population cross type, (i.e. whether family crosses were made within or between populations) was also not found to significantly improve the model fit (in comparison to a model including only sire, dam, and sire x dam effects) in the analysis of hatching time in either the control (LRT: χ^2^ = 2.3, p = 0.13) or the treated group (LRT: χ^2^ = 0.45, p = 0.50). Similarly, population cross was not important in terms of hatching size or yolk sac volume in neither the control (LRT: χ^2^ = 0.06, p = 0.80; χ^2^ = 2.0, p = 0.16) nor the *P. fluorescens* treatment (LRT: χ^2^ = 0.11, p = 0.73; χ^2^ = 2.9, p = 0.09). We also did not find any indication of an interaction between population cross and treatment for any trait (Table 1, Figure 4). Post-hoc power analysis revealed that our power to detect differences between population cross types in the expression of larval traits in different environments was rather low. Power to detect this effect for hatching time in the control and bacteria-treated groups was, respectively, equal to 0.32 and 0.11. Power to detect differences between hybrid- and within-population crosses in larval length and yolk sac volume was, respectively, equal to 0.04 and 0.35 in the control group, and 0.05 and 0.53 in the *Pseudomonas* treatment. Similarly, our ability to detect interaction effects between population cross type and treatment was low for all traits (hatching time: 0.32; larval length: 0.04; yolk sac volume: 0.08).

Dam effects were significant across treatments for every examined trait (Table 3). Sire effects also accounted for significant variation in all traits and treatments (Table 3). Additive genetic variance tended to be slightly reduced in the pathogen treatment for hatching time and length. Dominance interaction effects never accounted for an important amount of variation. Heritability of hatching time was moderate to high, while that of the other early life history traits was low.

**Table 3:** REML estimates of variance components (V_A_: additive genetic; V_Dam_: dam; V_M_: maternal environmental; V_D_: dominance genetic; V_Res_: residual) for hatching time, larval length, and yolk sac volume for each treatment, in addition to heritabilities and coefficients of additive genetic variation (CV_A_).

	Hatching Time		Length		Yolk Sac Volume	
	Control	PF	Control	PF	Control	PF
V_A_	74.8***	45.6***	0.002***	0.001^**^	4.8^*^	5.8^**^
V_D_	5.2	0	0	0	0	4.4
V_Dam_	58.9***	36.3***	0.002***	0.001***	172.5***	164.3***
V_M_	40.2	24.9	0.002	0.001	171.3	162.8
V_Block_	0	12.8	0.0003	0.0005	17.0	26.7
V_Residual_	105.1	92.6	0.01	0.01	49.4	37.1
*h* ^2^	0.40	0.30	0.13	0.07	0.02	0.03
CV_A_	1.9	1.4	3.7	2.8	1.3	1.5

Linear mixed models were fitted to the data independently by treatment for each trait, and variance components were extracted from the models (see Methods for details). The significance of each variance component was determined by comparing a model incorporating the effect of interest to one lacking it.

*** p<0.001, ^**^ p<0.01, ^*^ p<0.05, ^+^p<0.10.

**Figure 5:**
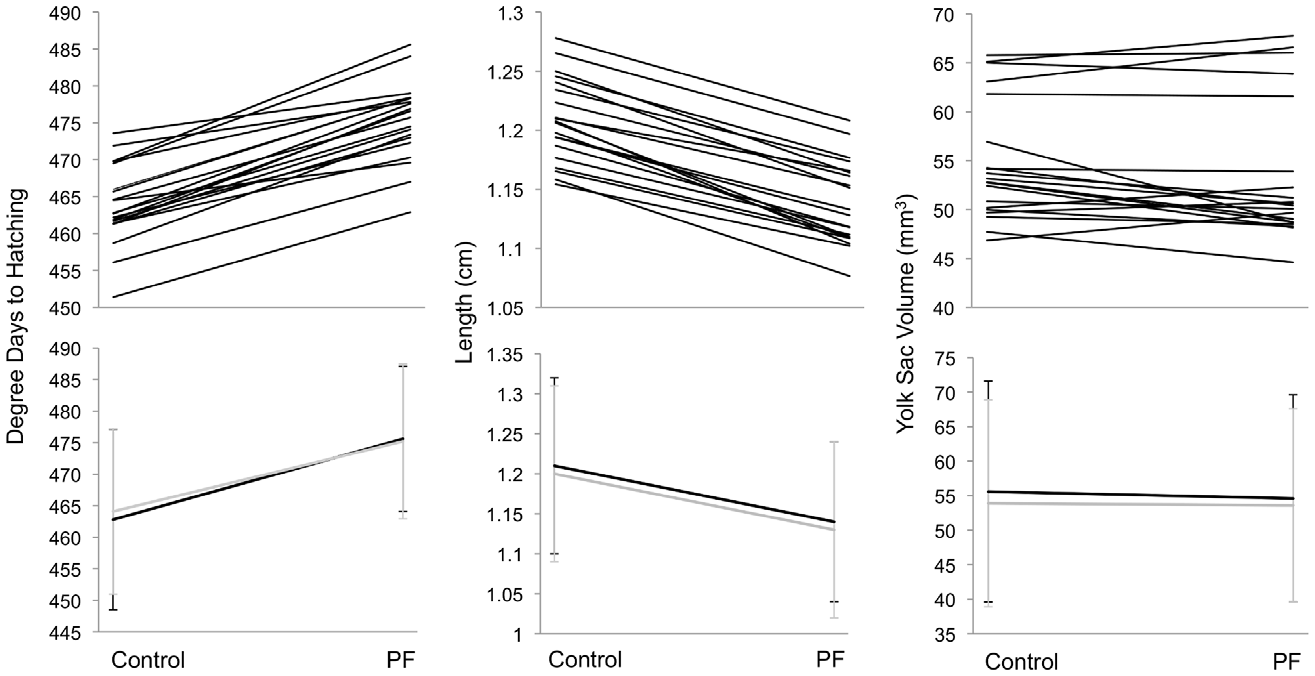
Reaction norm plots of hatching time, larvae length, and yolk sac volume. In the upper panel, each line corresponds to sire (N = 20) means across all females. Lines in the lower panel represent means (±SD) per within-population (black) or between-population cross (light grey). PF: *P. fluorescens*.

We observed significant genetic variation for hatching plasticity indicated by the s x t interaction (Table 1; Figure 5) but not for length or yolk sac reaction norms (Table 1). Treatment x dam effects were evident for hatching time and hatchling length, suggesting an interaction between maternal factors and treatment. The three-way interaction (t x s x d) was significant only for hatching time.

Egg size was not correlated to hatching time in either treatment (p always >0.05). Egg size was also not correlated to hatchling length in the control (r = 0.31, p = 0.19), or the pathogen treatment (r = 0.45, p = 0.05), after Bonferroni correction (α = 0.008). Egg size was always positively correlated to yolk sac volume (control: r = 0.93, p<0.001; *P. fluorescens*: r = 0.92, p<0.001). Hatching time was positively correlated across treatments (r = 0.81, p<0.001), as was hatchling length (r = 0.90, p<0.001) and yolk sac volume (r = 0.92, p<0.001).

## Discussion

We have crossed five genetically distinct populations of brown trout to investigate how outcrossing affects the expression of early life history traits under benign and stressful conditions. As we used a full-factorial breeding approach, we were able to disentangle the effects of genetic crossing distance from sire and dam effects, and examine how pathogen stress influences the importance of additive genetic, maternal environmental, and dominance interaction effects on fitness-related traits.

### Treatment effects on early life-history traits

Inoculation of brown trout embryos with *P. fluorescens* did not increase mortality in this experiment. In contrast, treatment with other strains of this bacterium was previously found to cause embryonic mortality in another salmonid (*Coregonus* sp.) [55], [67]. Notably, pathogen virulence is often strain specific [e.g. 68], [69], [70], [71]. Moreover, the above studies on whitefish described infections during later stages of embryonic development, and susceptibility is often age-dependent [72], [73], [74], [75], [76], possibly due to changes in host resistance to infection and/or tolerance to pathologies [77].

While inoculation did not increase mortality, it delayed hatching time and resulted in smaller hatchlings with reduced yolk sac volumes. Hatching time may be induced or delayed depending on whether the risk is specific to eggs or larvae [54], [78], and variation in hatchling size can reflect an adaptation to prevailing ecological conditions [11]. Given that these traits are plastic, the phenotype which best fits the environment should, in theory, be expressed [1]. However, the plasticity that we observed here may not reflect shifts in trait means towards environment-specific optima. Late hatching is often selected against in trouts and salmons (*Salmo* sp. and *Oncorhynchus* sp.), as it delays access to feeding territories [79]. Moreover, mortality during early life stages is size-selective, with larger fry being more resistant to starvation, and better able to evade predators and hunt larger prey items [20], [80]. The changes in phenotypes are, therefore, probably ‘negative direct effects’ [81] of infection, resulting from physiological constraints on trait expression [82]. Indeed, the reduced size of the larvae may reflect increased metabolic demands associated with an immune response [83], i.e. proteins in the yolk sac, e.g. antibodies, that could have served as sources of nutrition were instead used to fight infection [84].

### Effects of outcrossing distance on fitness

Theory predicts that genetic crossing distance has an effect on offspring fitness, with a viability peak at intermediate distances, where both the effects of inbreeding and outbreeding depression are the least [34], [85]. We found no evidence of a relationship between genetic crossing distance and phenotype here. Moreover, we found no indications of a relationship between the relatedness of breeders and phenotype. We also found that outcrossing, with respect to within vs. between-population crosses, had no apparent effect on fitness-related traits in terms of either phenotypic means or reaction norms. In contrast, Darwish and Hutchings [22] found significant changes in life-history reaction norms in second generation hybrids of Atlantic salmon (*S. salar*) when introgressed with either genes from another wild population, or those belonging to captive populations. Fraser et al. [23] observed that hybridization between wild and farmed Atlantic salmon resulted in decreased survival at low pH and maladaptive changes in reaction norms. A salient feature of their study was that lowered fitness was only apparent in the F_1_ generation, with backcrosses and F_2_ hybrids performing as well as wild crosses.

A few factors may explain the general uniformity between within- and between-population crosses in their reaction to the pathogen stressor. First, the overall genetic differentiation among the five populations was rather small [39]. Although the populations are significantly structured, with subpopulations both genetically and phenotypically (i.e. phenotypes were found to match flow regimes of respective habitats, see [39]) distinct, the overall range of genetic distances our crosses yielded may not have provided sufficient breadth to reveal inbreeding or outbreeding depression. This may have engendered low effect sizes, which would have given us limited power to detect differences between hybrid- and within-population crosses. Second, our study assessed the impact of population mixing on early life-history traits in F1 crosses, and although a number of studies have demonstrated decreased fitness in first generation hybrids [e.g. 23], [29], [31], diminished fitness is often more apparent in or following the F2 generation [34]. Thirdly, although interpopulation crossing did not impact fitness-related traits during the time points that we examined, it is possible that it could become influential at later developmental stages, as genetic incompatibilities between parental genomes become apparent [27]. For example, Leary et al. [25] found that first generation hybrids of rainbow trout (*O. mykiss*) and westslope cutthroat trout (*O. clarki lewisi*) showed signs of hybrid vigor until hatching, after which mortality sharply increased in comparison to within population crosses.

### Genetic organization of early life-history traits across environments

Time to hatching, hatchling length, and yolk sac reserves are all traits that are tightly linked to fitness, and as such, they are expected have reduced genetic variation because of directional selection [86]. The genetic variability in these traits is instead anticipated to be largely maintained by dominance interaction effects [87]. However, we found little evidence of non-additive genetic variance, and rather observed that these traits harbored significant additive genetic variance. Significant genetic variability in fitness traits was previously found in brown trout [56], [57], [88] and other species [e.g. 19], [65]. Comparatively, both hatchling length and yolk sac volume appeared to have lower heritabilities than hatching time; however, this seemed attributable to the larger residual variances in these traits-a factor that often leads to the underestimation of the amount of genetic variability in a character [89].

Interestingly, we found no evidence of stress-dependent effects on the expression of genetic variability in any of the traits, despite the fact that treatment resulted in significant changes in hatching time, larval length, and yolk sac reserves. Our findings are consistent with other studies that have found no pronounced effect of stress on heritable variation in traits [17], [90]. Notably, stress-dependent changes on additive genetic variation are common [15], so our results simply highlight that fact that the effect may be dependent on both the trait in question, as well as the stressor.

Like additive genetic variance, maternal effects were also consistently significant, which is to be expected for early life-history traits [91]. Notably, their magnitudes appeared smaller than that of genetic effects for hatching time, were comparable for hatchling length, and were much larger for yolk sac volume, suggesting that their relative importance was somewhat trait-dependent. In the case of hatching age, the maternal effects appeared largely independent of egg size. On the contrary, egg size was highly correlated with yolk sac reserves across environments. Egg size, while not always an indicator of quality [12], has been linked to increased fitness (i.e. survival and growth) in Chinook salmon (*O. tshawytscha*) [91] and brown trout [80]. The consequences of maternal effects also appeared to vary according to the treatment for hatching time and hatchling length, as suggested by the dam by treatment effects. Einum and Fleming [80] similarly demonstrated that adaptive value of maternal traits can change with the environment.

### Genetic variation for trait plasticity and cross environment correlations

Although the plastic responses we observed after pathogen challenge may not have been strictly adaptive, this does not exclude the possibility that trait plasticities are beneficial. For instance, certain genotypes may be better adapted to the stressor and more capable of achieving optimal phenotypes [81]. Like other studies on plasticity in salmonids [reviewed in: 8], we found evidence of genetic variation for hatching age reaction norms; however, we found no such indication for hatchling length or yolk sac volume. At the same time, we found that all traits in question were correlated across environments. Cross-environmental correlations imply that trait expression is mediated by the same loci in both settings [92]. Consequently, traits cannot evolve independently, and the evolutionary potential of the reaction norm is constrained.

## Conclusions

Treatment of brown trout embryos with *P. fluorescens* did not increase embryonic mortality, but decreased fitness in that it delayed hatching time, and resulted in smaller larvae with diminished yolk sac reserves. Our results, therefore, indicate that a multi-trait approach may be necessary in assessing the virulence of a given pathogen. Contrary to expectations, we found no evidence of a relationship between genetic distance and phenotype, and also observed that within vs. between-population crosses did not differ significantly in phenotypic means or reaction norms. However, we do not wish to suggest that population mixing is always benign. Deleterious effects may come to light at larger genetic distance between the populations. Moreover, even if embryos are sensitive indicators of genetic or environmental problems [e.g. 88], [93], [94], [95], [96], [97], noxious effects at later developmental stages or in later generations cannot be excluded. Finally, we did not find evidence of stress-dependent changes in the expression of genetic variability, an effect that may be contingent on both the trait and stress in question.
